# Research Progress of the Gut Microbiome in Hybrid Fish

**DOI:** 10.3390/microorganisms10050891

**Published:** 2022-04-24

**Authors:** Xinyuan Cui, Qinrong Zhang, Qunde Zhang, Yongyong Zhang, Hua Chen, Guoqi Liu, Lifeng Zhu

**Affiliations:** 1 College of Life Sciences, Nanjing Normal University, Nanjing 210046, China; cuixinyuanwj@163.com (X.C.); z1187438993@163.com (Q.Z.); qundezhang23@163.com (Q.Z.); zyy863098363@163.com (Y.Z.); 2 Mingke Biotechnology, Hangzhou 310000, China; chenhua@mingkebio.com (H.C.); liuguoqi@mingkebio.com (G.L.)

**Keywords:** hybrid fishes, gut microbiome, community and function, speciation, invasion, fish conservation and management

## Abstract

Fish, including hybrid species, are essential components of aquaculture, and the gut microbiome plays a vital role in fish growth, behavior, digestion, and immune health. The gut microbiome can be affected by various internal and/or external factors, such as host development, diet, and environment. We reviewed the effects of diet and dietary supplements on intestinal microorganisms in hybrid fish and the difference in the gut microbiome between the hybrid and their hybrids that originate. Then, we summarized the role of the gut microbiome in the speciation and ecological invasion of hybrid fish. Finally, we discussed possible future studies on the gut microbiome in hybrid fish, including the potential interaction with environmental microbiomes, the effects of the gut microbiome on population expansion, and fish conservation and management.

## 1. Introduction

The host and its microbiome are regarded as a unique biological entity holobiont, including the genome, which is called the hologenome [1]. The combination of complex microbiota and genes in the intestine are collectively referred to as the gut microbiome [2]. Animal hosts maintain a long, close, and complex relationship with their gut microbiome [3]. The gut microbiome plays a vital role in the nervous system development [4], behavior [5], immunity [6], food digestion, and metabolism [7] of the host. Gut microbiota are highly specialized microbial communities with a complex composition that is affected by many interactions among microorganisms, host, diet, and the environment [8]. Host phylogeny and diet are the two main factors shaping the animal gut microbiome [9,10,11,12,13,14].

Fish comprise nearly 50% of the total vertebrate diversity, and more than 34,000 species have been described to date, constituting a crucial part of the aquatic ecosystem [15,16]. Microorganisms exist in almost every fish organ, including the skin, digestive tract, internal organs, and luminous organs [17]. The fish gut is a complex ecosystem, composed of highly diverse microbiota. The microbiota is influenced by various factors, such as habitat environmental factors, season, host genetics, developmental stage, nutrition level, and diet composition, with the potential major determinant being the habitat environment [16].

Overall, bacteria are the primary microbial colonizers in the gastrointestinal tract of fish [18,19,20,21]. The gastrointestinal microbiota of fish mainly consist of aerobic or facultative anaerobic microorganisms and facultative and obligate anaerobes [20,22,23,24]. Among them, Proteobacteria, Firmicutes, and Bacteroidetes constitute 90% of the gut microbiome of most fish [15]. In addition, Actinobacteria, Fusobacteria, Bacilli, Clostridia, and Verrucomicrobia are the dominant bacterial phyla in fish gut microorganisms [15,25,26,27,28,29]. The gut microbiota of fish participate in various physiological functions. There are several beneficial effects on the host, such as reproduction, development, nutrition, immunity, and stress responses, and the gut microbiota are often referred to as an ‘extra organ’ [15,30]. Nayak has described the role of fish gastrointestinal microbiota in nutrition, immunity, and health management [20].

Early research on fish gut microflora employed culture-dependent techniques. The emergence of metagenomics and next-generation sequencing techniques has entirely changed fish gut microbiome research by presenting a method that directly analyzes the microbial genome from environmental samples [31,32]. These new research methods have led to a better understanding of the connections between the microorganisms and their respective hosts. The Illumina system, Roche 454 system, and Ion Torrent Personal Genome Machine (PGM) are the primary next-generation sequencing (NGS) platforms used in fish gut microbiome research, and the Illumina system is the most commonly used [15].

The influencing factors and physiological functions of fish intestinal microbiota are two critical issues in NGS analyses [33]. Most studies have explored the effects of various host and environmental factors on the bacterial community composition of gut microbiota. Limited studies have analyzed the beneficial and harmful effects of the gut microbiota on the host [15]. However, there are many valuable bacterial species in the intestines of fish, including *Cetobacterium* spp. and *Lactobacillus* spp. [34]. Hybrid fish are indispensable components of fish species and are essential in aquaculture. We review recent research on the gut microbiome and ecological problems in hybrid fishes and discuss possible future research to improve our understanding of the gut microbiome in fish.

## 2. The Gut Microbiome in Hybrid Fish

### 2.1. Effects of Diet and Dietary Supplements on the Gut Microbiome and Immune Health of Hybrid Fish

Hybridization is a basic step in the long-term evolution of organisms, which may lead to the production of new species. Heterosis is a complex biological phenomenon where the hybrid offspring show superior natural characteristics, when compared with their parents [35,36]. Heterosis occurs in fish, and hybrid fish have advantages of faster growth performance, higher immunity, improved ecological adaptability, and an enhanced tolerance for transportation. Therefore, as wild catch fisheries can no longer support the world consumption of seafood, fish heterosis has been widely assisting aquaculture since the 1980s [37,38,39,40,41]. However, even the improvements made by heterosis may not be enough for the growing world consumption rate of fish [35]. 

In addition, different fish species inhabiting the same waters may also naturally hybridize in the wild. Hybrid fish may possess improved ecological adaptability compared to their parents and be more widely distributed in the natural environment with heterosis, due to the survival of the fittest theory [42,43,44]. In reality, the microbiota in hybrids may provide new favorable physiological functions and promote the utilization of new ecological niches, and the hybrid microbiota may also shape reproductive barriers, which may influence the ecological speciation or the expansion of the population range [45,46,47,48]. It has been shown that greater than 30,000 variations of hybrid fish species have formed in the wild, and these large fish populations can produce high diversity in the dietary niches. Therefore, exploring the microbiota of wild hybrid fish is of great significance for understanding the basic biological and ecological processes of speciation, population expansion, and invasion ecology [39].

We, firstly, aimed to provide a whole picture of the diet or dietary supplement effects on the fish gut microbiome (Table 1) [15,20,49,50]. Then, we focused on the relationship between the diet and the hybrid fish gut microbiome. We found that many studies have explored changing the diet or dietary additives on the composition and function of the hybrid fish gut microbiome and their promotion of the growth and health of mixed fish (Table 2), but rare in the comparison between the hybrid and their hybrids’ origin.

#### 2.1.1. Antibiotics

Infectious diseases caused by various pathogens have severely harmed the health of aquatic organisms around the world [125]. Antibiotics have been widely used as feed supplements to treat intestinal diseases in fish and have become indispensable in human health [33,126,127]. A short-term (6 days) dietary antibiotic mixture (vancomycin, neomycin sulfate, and metronidazole) can improve the lipid metabolism in hybrid groupers (*Epinephelus fuscoguttatus* ♀ × *E. lanceolatus* ♂) fed medium- and high-lipid diets. However, antibiotic treatments can also strongly alter intestinal microbiota by reducing the relative abundance and diversity of hybrid grouper gut microbiota, resulting in a significant increase in the proportion of Bacteroidetes and a decrease in the proportion of Firmicutes [93]. Long-term antibiotic supplementation can cause several side effects on fish health [127,128,129]. Presently, the pollution and spread of antibiotic-resistant genes caused by the long-term abuse of antibiotics have become a global problem [130]. Recently, probiotics and prebiotics are an emerging strategic approach for sustainable aquaculture, as they do not cause environmental pollution or public health hazards [51,131,132].

#### 2.1.2. Probiotics

Probiotics are beneficial microorganisms that can modulate intestinal microbial composition and improve the host health status [133,134]. Probiotics are commonly used in the aquaculture industry as feed or water additives [20]. The essential probiotic microorganisms employed in aquaculture are lactic acid bacteria (LAB) species [135,136] and *Bacillus spp*. [52,137]. The other general probiotic species used in fish are *Saccharomyces*, *Clostridium*, *Enterococcus*, *Shewanella*, *Leuconostoc*, *Lactococcus*, *Carnobacterium*, and *Aeromonas* [20]. Fish are vulnerable to various pathogenic microorganisms, and innate immunity provides an initial line of defense [138]. The addition of probiotics to the diet plays a vital role in stimulating fish immune responses, and further promotes the innate and adaptive immune system [139]. For an example, *Bacillus subtilis* strain 7k, isolated from the gastrointestinal tract of hybrid hulong grouper (*Epinephelus fuscoguttatus* × *E. lanceolatus*), could be used in grouper culture to stimulate growth, enhance immunity and promote health in the fishes [94]. Studies reveal that *O. mykiss* fed different types of probiotics increased the expression of the TGF-β gene, which regulates fish immunity [140,141,142]. TGF-β levels increased in juvenile hybrid tilapia (*O. niloticus* ♀ × *Oreochromis aureus* ♂), after consuming a diet supplemented with *Bacillus subtilis* C-3102 [95], and the same occurred in Koi carp (*Cyprinus carpio*) [143]. HWF™ is a paraprobiotic and postbiotic supplementary diet using inactive and beneficial bacteria, and is considered an efficient therapeutic agent in fish. Feeding hybrid sturgeons (*Acipenser baerii* × *Acipensers chrenckii*) with HWF™ improved their growth and immunity by changing the composition and diversity of the gut bacteria, developing their healthy gut microbiota [96].

#### 2.1.3. Prebiotics

Prebiotics are an innovative strategy, providing a dietary supplement to improve growth development and the immune system by regulating gut microbiota [144]. Prebiotics are generally non-digestible oligosaccharides added to fish feed as dietary components to promote the proliferation of specific beneficial microorganisms in the intestine and, thus, enhance host health [145]. Previous research has shown that prebiotics can decrease the adherence and colonization of pathogenic microorganisms in the intestinal tract to improve the general immunity of the host by increasing the number of lactic acid bacteria, especially *Bifidobacterium* [20,146,147]. Fructo-oligosaccharides, galactooligosaccharides, mannan-oligosaccharides (MOS), xylooligosaccharides (XOS), inulin, lactulose, and lactosucrose are common prebiotics used in various animals, including humans [20]. The level of gut lactic acid bacteria was significantly increased in hybrid catfish (*Pangasianodon gigas* × *Pangasianodon hypophthalmus*) fed with diets containing 0.6% xylooligosaccharides (XOS) [97]. In addition, several studies have reported that inulin, fructooligosaccharides, xylooligosaccharides, galactooligosaccharides, and arabinoxylan-oligosaccharides can affect growth development, immune health, and the composition and/or diversity of the gut microbiota in different fish species [53,97,148,149,150,151]. Indeed, many researchers have reported the effect of prebiotics on the gut microbiota in fish, such as grass carp [54], Siberian sturgeon [53], Nile tilapia [55], and European sea bass [152].

The prebiotic Grobiotic™AE and dietary brewer’s yeast can improve the growth performance, immune response, and resistance to *Streptococcus iniae* infection in hybrid striped bass (*Morone chrysops* × *M. saxatilis*) [153]. Dietary supplementation of 4% ESTAQUA^®^ yeast culture (YC) for hybrid grouper (*Epinephelus fuscoguttatus* ♀ × *E. lanceolatus* ♂) could improve the alpha diversity of gut microbiota, growth performance and serum immune responses against *V. harveyi* attacks [98]. N.B.T. is an excellent indicator of the health status and/or immunization effectiveness in fish [56]. Supplementing the diet with raffinose in hybrid sturgeons (*Acipenser baeri* Brandt ♀ *× A. schrenckii* Brandt ♂) improved the growth performance and intestinal morphology, modifying the gut microbiota composition and increasing the level of N.B.T. activity [99]. Chitosan oligosaccharide (COS) is a new prebiotic, dietary COS supplementation, which improves the growth performance and health status of *Scopthalmus maximus* [154], *Cyprinus carpio koi* [155], and *Oncorhynchus mykiss* [156]. Dietary COS supplementation improved the intestinal health and immune responses of hybrid groupers (*Epinephelus fuscoguttatus* ♀ × *E. lanceolatus* ♂) when fed a low-fish meal diet [100].

It is worth noting that prebiotic supplementation is only beneficial when a moderate volume is provided; prebiotics at a high concentration can be harmful to the host. Excessive prebiotics may cause an imbalance in the gut microenvironment, which decreases the digestive capacity in fish intestines. A previous study revealed that a high concentration of inulin could damage the enterocytes of *Salvelinus alpinus* [157]. This may explain why 0.4–0.6% COS supplementation was optimum in hybrid groupers [100].

#### 2.1.4. Fishmeal Protein Substitutes

Fishmeal (F.M.) is the most widely utilized high-quality protein source in aquatic feed and has many advantages [158]. However, fishmeal production cannot meet the growing needs of the aquaculture industry due to its rapid development, which is causing a severe impediment to industry development [101,159]. Therefore, using plant proteins is an innovative solution for sustainable aquaculture [160,161].

Cottonseed protein concentrate (CPC) is a new experimental fishmeal (FM) replacement [162]. However, fishmeal replaced with CPC in an inappropriate proportion can have adverse effects on the intestinal health of groupers and leads to intestinal inflammation [163]. A study on pearl gentian groupers (*Epinephelus fuscoguttatus* ♀ × *Epinephelus lanceolate* ♂) revealed that 24% CPC was considered the most appropriate volume for F.M. replacement and growth performance, digestive proteinase activity, intestinal morphology, and intestinal microflora in the pearl gentian grouper reached maximum levels with 24% CPC replacement levels. Subsequently, many physiological parameters are reduced with increasing CPC replacement levels [101]. The substitution of FM with peanut meal (PNM) of up to 50% or CPC up to 60% obviously changed the intestinal microbiota of juvenile hybrid groupers *(E. fuscoguttatus* ♀ × *E. lanceolatus* ♂), which increased intestinal pathogenic bacteria and decreased intestinal beneficial bacteria [102,103]. Similarly, replacing FM with peptides from swine blood (PSB) up to 75% could reduce growth performance for hybrid groupers (*Epinephelus fuscoguttatus* ♀ × *E. lanceolatus* ♂), and increase the abundance of the potentially pathogenic *Pseudomonas* and *Arcobacter* in the gut [104].

Another fishmeal replacement protein is soybean meal (SBM). SBM has been widely considered an inexpensive FM replacement [164]. Nevertheless, anti-nutritional factors in SBM can negatively affect the intestinal morphology of fish [165]. Research reveals that bioprocesses (such as soybean meal ingredients) can reduce the intestinal microorganism diversity in hybrid striped bass (*Morone chrysops* × *M. saxatilis*) [105]. It is challenging to find a suitable fish meal substitute for various fish, and protein substitutes have excellent potential and are important future research topics.

### 2.2. Hybrid Speciation and Gut Microbiome

No living organisms exist in isolation from the microbial world, and microbial symbiosis and speciation profoundly shape the biodiversity composition. Animal hosts and microbiomes are closely interconnected and interact over long evolutionary timeframes. They can even be regarded as a unique biological entity-holobiont and include their entire genome, called the hologenome [1]. Diverse and complex interactions exist between hosts and microorganisms. Microorganisms play essential roles in host physiology, health, and survival. Microorganisms can even alter host reproduction [166], resulting in host embryo death [167,168,169,170] and affect the host gametic integrity and embryonic viability, which may be closely related to the formation of new species [45,171]. The microorganisms and their interactions with hosts are potentially important factors in stimulating the formation of new species [172].

Species are reproductively isolated groups composed of potentially interbreeding individuals, and hybrids can suffer from post-mating isolation barriers, such as sterility and/or unviability [173]. The composition and functional effects of animal microbiota are closely related to host evolution, and the survival rate and performance of microorganisms can be reduced when interspecific microbiota transplantation occurs between closely related and different host species pairs. The microbiome compositional relationships (i.e., beta diversity) reflect the evolutionary relationships of the host species [173,174]. Thus, natural selection can drive phylosymbiotic changes within the parental species, which may lead to the evolution of deleterious interactions between hybrids and their microbiomes [173].

Based on the holobiont concept, host-genome–microbiome associations and their role in host adaptability demonstrate that microorganisms may participate in the process of speciation, and symbiotic microorganisms may hinder speciation through isolation, including behavioral isolation, geographical isolation, and reproductive isolation [45]. Microbial symbionts can add new functional genes to the host genome, which assists the host in expanding its dietary niche and obtaining new nutritional opportunities. Unfortunately, hybridization can inhibit symbiotic relationships by destroying the vertical transmission of some microorganisms between the host parents and offspring, which are hybridization disadvantages and hinder species formation, as observed in *Acyrthosiphon pisum* [175], *Sitophilus* [176] and the family *Plataspidae* [177]. In hybrid species, microorganisms can hinder speciation by assisting reproductive isolation. *Wolbachia* is a bacterium that widely exists in the reproductive system of arthropods and may cause hybrid male sterility in *Drosophila paulistorum* [178]. In the two-spotted mite (*Tetranychus urticae*), *Wolbachia* can also cause cytoplasmic incompatibility (CI) in the F1 generation and F2 male offspring deaths from the surviving F1 females in the CI cross [178]. Similarly, different CI Wolbachia in *Nasonia* wasp species can cause high levels of F1 hybrid lethality and the reproductive isolation induced by CI has evolutionary potential in the early stages of the speciation process [179,180].

Similarly, a close interaction exists between the gut microbiome and host, and plays an important role in the speciation of hybrid species. For example, the host gut microbiome may hinder the formation of new species by participating in the death of hybrids in *Nasonia* wasp species [181]. Vertebrates are a vital group for interactions in reproductive isolation and speciation research. Alterations in gut microbiota communities and increases in gut pathology exist in hybrid mice *(Mus musculus* × *Mus domesticus*) [46]. The gut microbiome does not always play negative roles in hybrid species. For example, the hybrid offspring of sika deer (*Cervus nippon*) and elk (*Cervus elaphus*) harbor a high abundance of *Acetitomaculum* bacterial species, which may assist in the absorption and metabolism of nutrients [182,183]. A similar phenomenon was identified in the hybrid offspring of ponies and donkeys, which render a completely different gut microbiota from their parents [184].

In the gut microbiome in hybrid fish research, differences in the gut microbiome between hybrid offspring and parents have been observed. In lake whitefish (*Coregonus clupeaformis*), the gut microbiome is significantly different between the F1 hybrids and their parents, especially the abundance difference between Firmicutes and Proteobacteria [106]. The research also found the interactions of the host-microbiota-environment demonstrated three different evolutionary paths in the gut microbiome [106]. Similarly, the gut characteristics of hybrid fish from herbivorous blunt snout bream (*Megalobrama amblycephala*) and carnivorous topmouth culter (*Culter alburnus*) differ from their parents. The microbial community in the hybrid topmouth culters was markedly distinct from their parents, and varied in the cellulose content in the gut [39]. One study found that the evolutionary characteristics of hybrid fish progeny from *Megalobrama amblycephala* and *Culter alburnus* may be manifested in dietary adaptation and choice; the interactions between gut microbiota and host genetics contributed to hybrid fishes adapting to herbivorous diets more than carnivorous diets [185]. Compared to the parents, the hybrid offspring of two invasive North American carp, *Hypophthalmichthys nobilis* and *Hypophthalmichthys molitrix*, harbor different gut microbiome compositions and display higher alpha diversity than their parents [107].

### 2.3. The Differences in the Gut Microbiome of the Hybrid Fish and Their Hybrids Origin

There are still few studies directly comparing gut microbiome between parental and hybrid progeny. However, it has been shown that existing differences in intestinal microbiota between captive parents and hybrid fishes’ offspring exist under a controlled environment [106]. There is no doubt that diet will affect the gut microbiome composition and growth performance of the host, and under the same dietary conditions (Artemia and mixed diet), the taxonomic composition of transient gut microbiota between both whitefish (*Coregonus clupeaformis*) parental species and their reciprocal hybrids showed a slight pattern of differentiation, which, within the Artemia diet group, meant a higher abundance for Firmicutes, but lower for Proteobacteria, was observed in hybrids in comparison with their parents’ whitefish, while the opposite result was found in the mixed diet group, where there was a higher abundance of Proteobacteria but it was lower for Firmicutes. In addition, in the abundance composition of some specific bacterial genera, the two reciprocal hybrids, and their parents also showed the opposite pattern, that F1 D♀N♂ has more specific bacterial genera than its parents, while F1 N♀D♂ with fewer specific bacterial genera than its parents. In the hybridization experiment between whitefish and omul (*Coregonus migratorius*), the researchers found that the hybrid progeny had a lower alpha diversity (e.g., Shannon index) in hindgut microbiota than the parents [186].

Host genetics can strongly affect the gut microbial composition of the hybrid offspring [39]. Compared with carnivorous topmouth culter (*Culter alburnus*, TC) parents, the gut microbiome structure of their two-hybrid progenies is more similar to that of herbivorous blunt snout bream (*Megalobrama amblycephala*, BSB) parents, as the alpha diversity of the two types of hybrids and BSB parent is higher than that of a TC parent, as well as beta diversity analysis, which also showed that there was no significant difference between the two hybrids and the BSB parent. Interestingly, in the composition of gut microbiota, Fusobacteria and Proteobacteria are the most abundant intestinal flora in hybrid fishes, and the proportion of Fusobacteria and Proteobacteria in hybrid offspring is similar to the BSB parent but significantly different from the TC parent. Again, the shared bacterial taxa at the phylum level showed different results; the hybrids of the two types share higher proportions of gut bacterial communities with the BSB parent than the TC parent.

Recently, our study reported a direct comparison of the similarities and differences in gut microbiome (composition and potential function) among bighead carps (*Hypophthalmichthys nobilis*, B), silver carps (*Hypophthalmichthys molitr*, S) and their hybrid offspring (SB and BS) in ponding experiments [107]. The hybrid gut microbiome displays the admixed pattern at the community level and harbors the relatively high alpha diversity (e.g., phylogenetic diversity). For example, the hybrid fish had intermediate abundances of Cyanobacteria and Bacteroidetes in the foregut, while Fusobacteria are significantly enriched in parents in the hindgut. Moreover, the hybrid gut microbiome’s predicted function shows the enrichment in the genes coding for putative enzymes involved the diet utilization, which suggests the potential benefits to their local adaptation. 

### 2.4. Gut Microbiome Might Promote Ecological Invasion by Hybrid Fish

Gut microbiota can enhance the adaptability of the host to the environment and improve the successful invasion rate of some invasive species [187]. For invasion success, the species requires a dispersal ability, environmental tolerance, phenotypic plasticity, and associated epigenetics [188,189]. Host shifts can lead to phytophagous insects becoming invasive species [190]. It has already been demonstrated that the gut microbiome plays a vital role in phytophagous insect invasion success [191], and gut bacteria can assist in the successful invasion of insect species by regulating epigenetic factors related to the host [192]. Similarly, some biological mechanisms can enhance the success rate of invasive species, such as genetic diversity [193], reproductive rate [194], food resources [195], and hybridization [44,196].

Therefore, there are complex and close relationships between hybridization, the gut microbiome, and bio-invasion. Bighead carp and silver carp are invasive species, characterized by various hybridization in the Mississippi River Basin [107]. There is higher alpha diversity in the foregut microbiota in the hybrid offspring, and an increasing discrepancy also occurs between the foregut and hindgut. Similarly, the hybrids had a higher proportion of putative genes coding for putative enzymes related to the digestion of filter-feeding phytoplankton (Cyanobacteria, cellulose, and chitin) than their parents. The improved putative enzymes could encourage the utilization of new food resources by the gut microbiota and, therefore, improve survival, environmental adaptation, and invasion by hybrid fish. Therefore, the gut microbiome and host genome may synergistically promote bigheaded carp invasion in the United States [107].

## 3. The Potential Impact of Environmental Microbiota

The current research focuses on fish, not hybrid fish. However, environmental microbiota impacts may also occur in hybrid fish.

### 3.1. Habitat Environmental Microbiome Shapes the Early Gut Microbiome of Juvenile Fish

The main determinant of fish gut microbiota is the natural environment, and fish intestinal microbiota symbionts are generally obtained from the environment [197] by neutral processes, such as drift and diffusion, which produce most of the microbial diversity [198]. The microorganisms transmitted from the environment to the fish intestine are mainly derived from two paths: the foodborne microorganisms carried by prey and the microorganisms in the water, and most of the environmental microbiota remain temporarily in the fish gut [199]. In most fish species, the ontogeny and colonization of gut microbiota in the early stages of life rely on the horizontal transmission of environmental microbiota [200]. Juvenile zebrafish (*Danio rerio*) acquire gut symbiotic bacteria from the water environment after hatching, which may promote the development and function of their intestines [201]. Similar patterns are observed in wild Atlantic salmon (*Salmo salar*), discus (*Symphysodon aequifasciata*) [200], grass carp (*Ctenopharyngodon idellus*), Mucha perch (*Siniperca chuatsi*), and southern catfish (*Silurus meridionalis*). The composition of the gut microbiota community of juvenile fish was more similar to the habitat water environment than the adults [197,200]. However, fish gut microbiota often differ from their surrounding environment after becoming adults [202]. Therefore, environmental microorganisms play an important role in shaping the gut microbiota in the early juvenile fish stages and, as fish mature, the environmental factors are less influential because the gut microbiota gradually differentiate from the environmental microbiota, showing individual variations [197,203].

### 3.2. Do Fish Specifically Select Proteus from the Water Environment?

The gut microbiota of fish are mainly Proteobacteria and Firmicutes, whereas amphibians, reptiles, birds, and mammals contain mainly Firmicutes and Bacteroidetes. The excessive reproduction and presence of *Proteus* may be a sign of ecological imbalance in the gut microbial community of mammals [204], as many symbiotic *Proteus* bacteria can translate into pathogens, and infect and promote inflammation in the host under specific conditions. Many studies have demonstrated that, regardless of the fish living environment, the gut microbiome is composed of a common core microbiome [205]. Major environmental microorganisms are rarely observed in fish intestines [49]. *Proteus* dominate the gut microbiota of most fish species [206]. The *Proteus* abundance can increase with the growth and nutritional level of the fish (from herbivorous to carnivorous). Conversely, the abundance of Firmicutes usually decreases with increasing nutritional levels [202]. The gut microbiome not only reflects the microorganisms in its surrounding environment but also characterizes the specific selection of the environmental microbiome by the host in grass carp (*Ctenopharyngodon idellus*) [207], silver Prussian carp (*Carassius auratus gibelio*) [208], and zebrafish (*Danio rerio*) [209]. Notably, the higher proportion of *Proteus* in the fish intestines indicates the fish host has specifically selected *Proteus* from the habitat water or *Proteus* has outperformed the other environmental bacterial taxa in the water. This discrepancy is an urgent problem needing to be explored [16]. 

## 4. Future Perspectives

**Figure 1:**
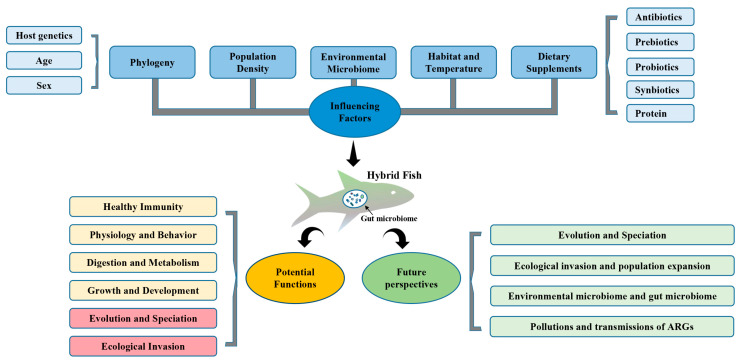
Major research progress and future perspectives on hybrid fish gut microbiome.

The gut microbiome can promote the successful ecological invasion of hybrid fish, which makes them occupy favorable ecological niches and further improves the potential for population expansion. Following Darwin’s theory of evolution, this process greatly improves the potential of hybrid fish to evolve into new species in the future (Figure 1). The gut microbiome plays a role in speciation, but its degree of impact remains unclear. Furthermore, the high genomic similarity between bighead and silver carp, and an over 90% embryonic viability in all crosses, indicate that interspecific hybridization between the carps might have promoted their range expansion [44]. In the future, the role of the gut microbiome in population expansion of hybrid species should not be ignored. It is highly significant for us to better combine the genome and metagenome to improve our understanding of the ecological problems of hybrid fish. The fish gut flora and fecal materials discharged into the water may reflect their diet preferences, physiological behaviors, and presence in the river [210], allowing gut microbiota to potentially monitor fish invasion and population expansion, which is an important research issue in fish conservation and management in the future (Figure 1).

For a long time, the source of gut microbiota has been an attractive research topic. Environmental microbiome transmission plays an important role in animal gut microbiota, and the differences between terrestrial and aquatic environments cause the gut of aquatic animals to be very different from that of terrestrial organisms, including fish and aquatic mammals. Research shows 13% of the gut microbiota of threespine stickleback (*Gasterosteus aculeatus*) comes from the surrounding water environment and 73% from prey [199]. In addition, in most fish species, the ontogeny and colonization by gut microbiota in the early stages of life mainly occur through the horizontal transmission of environmental microbiota [200]. Juvenile zebrafish (*Danio rerio*) acquire gut symbiotic bacteria from the water environment after hatching, potentially promoting the development and function of the intestines [201]. Similar patterns are observed in wild Atlantic salmon (*Salmo salar*), discus (*Symphysodon aequifasciata*) [200], grass carp (*Ctenopharyngodon idellus*), Mucha perch (*Siniperca chuatsi*), and southern catfish (*Silurus meridionalis*), and the composition of the gut microbiota community of juvenile fish was more similar to the habitat water than the adults [197,200]. In addition, different fish tissue types, such as skin, gills, and intestines, may also be the main determinants of microbiota diversity and composition [48]. Successful hybrid fish invasion depends on the relationships and interactions between an individual’s characteristics (age and gender), gut microbiome, environmental microbiome, and post-mating reproductive isolation, associated with environmental microbial transmission. Future research is required to assist our understanding of these interactions (Figure 1). In addition, the aquatic environment can become a reservoir of antibiotic-resistant genes (ARGs), providing an ideal path for the acquisition and dissemination of ARGs [211]. Aquatic animals, such as fish, are direct witnesses and victims of ARG-water pollution. Therefore, wild fish can be recipients and disseminators of ARGs in aquatic environments [130]. At present, there are few studies assessing ARG pollution and transmission in wild hybrid fish, providing great research potential in the future (Figure 1).

## Figures and Tables

**Table 1:** The application of diet and dietary supplements in fish.

Species	Class	Order	Family	Ingredients	Intervention Type	16s rRNA Sequencing	References
*Oreochromis niloticus*	Actinopteri	Cichliformes	Cichlidae	*Rummeliibacillus stabekisii*	Probiotic	Illumina MiSeq, Amplicon: V3–V4	[51]
*Oreochromis niloticus*	Actinopteri	Cichliformes	Cichlidae	*Bacillus subtilis*	Probiotic	Illumina HiSeq, Amplicon: V4	[52]
*Acipenser baerii*	Actinopteri	Acipenseriformes	Acipenseridae	Arabinoxylan-oligosaccharides (A.X.O.S.) + *Lactococcus lactis spp. lactis* or *Bacillus circulans*	Synbiotic	454 GS FLX Titanium, Amplicon	[53]
*Ctenopharyngodon idellus*	Actinopteri	Cypriniformes	Xenocyprididae	Xylo-oligosaccharide	Prebiotic	Illumina MiSeq	[54]
*Oreochromis niloticus*	Actinopteri	Cichliformes	Cichlidae	*Lactobacillus rhamnosus* JCM1136 and *Lactococcus lactis* subsp. *lactis* JCM5805	Probiotic	Illumina MiSeq, Amplicon: V3–V4	[55]
*Dicentrarchus labrax*	Actinopteri	Perciformes	Moronidae	Calcium carbonate	Prebiotic	Illumina MiSeq, Amplicon: V3–V4	[56]
*Ctenopharyngodon idellus*	Actinopteri	Cypriniformes	Xenocyprididae	*Bacillus subtilis*	Probiotic	No	[57]
*Ctenopharyngodon idellus*	Actinopteri	Cypriniformes	Xenocyprididae	*Bacillus coagulans*, *Rhodopseudomonas palustris* and *Lactobacillus acidophilus*	Probiotic	No	[58]
*Ctenopharyngodon idellus*	Actinopteri	Cypriniformes	Xenocyprididae	*Bacillus subtilis* Ch9	Probiotic	No	[59]
*Ctenopharyngodon idellus*	Actinopteri	Cypriniformes	Xenocyprididae	Exogenous cellulase	Prebiotic	Amplicon: V3	[60]
*Ctenopharyngodon idellus*	Actinopteri	Cypriniformes	Xenocyprididae	Glutathione	Prebiotic	No	[61]
*Ctenopharyngodon idellus*	Actinopteri	Cypriniformes	Xenocyprididae	*B. licheniformis* + xylo-oligosaccharide	Synbiotic	No	[62]
*Danio rerio*	Actinopteri	Cypriniformes	Danionidae	Gluten formulated diet	Protein	Illumina Miseq, Amplicon: V4	[63]
*Danio rerio*	Actinopteri	Cypriniformes	Danionidae	Protein meal of animal origin (*ragworm Nereis virens*)	Protein	454 GS FLX Titanium, Amplicon	[64]
*Danio rerio*	Actinopteri	Cypriniformes	Danionidae	Chitosan silver nanocomposites (CAgNCs)	Composites	454 GS FLX Titanium, Amplicon	[65]
*Gambusia affinis*	Actinopteri	Cyprinodontiformes	Poeciliidae	Rifampicin	Antibiotic	Illumina HiSeq, Amplicon: V4	[66]
*Oncorhynchus mykiss*	Actinopteri	Salmoniformes	Salmonidae	Dietary plant proteins	Protein	Illumina Miseq, Amplicon: V6–V8	[67]
*Oncorhynchus mykiss*	Actinopteri	Salmoniformes	Salmonidae	*Wickerhamomyces anomalus* + *Saccharomyces cerevisiae*	Synbiotic	Illumina HiSeq, Amplicon	[68]
*Oncorhynchus mykiss*	Actinopteri	Salmoniformes	Salmonidae	Microalgae meal (Schizochytrium limacinum)	Prebiotic	Illumina HiSeq, Amplicon	[69]
*Oreochromis niloticus*	Actinopteri	Cichliformes	Cichlidae	*Lactobacillus plantarum* CCFM8610	Probiotic	Illumina MiSeq, Amplicon: V4–V5	[70]
*Oreochromis niloticus*	Actinopteri	Cichliformes	Cichlidae	*Lactobacillus plantarum* CCFM639	Probiotic	Illumina MiSeq, Amplicon	[71]
*Oreochromis niloticus*	Actinopteri	Cichliformes	Cichlidae	*Vibrio sp.* CC8 and *Bacillus cereus* CC27,	Probiotic	No	[72]
*Oreochromis niloticus*	Actinopteri	Cichliformes	Cichlidae	*Clostridium butyricum*	Probiotic	Illumina HiSeq, Amplicon-	[73]
*Oreochromis niloticus*	Actinopteri	Cichliformes	Cichlidae	*Allium sativum*	Plant	Illumina MiSeq, Amplicon: V4–V5	[74]
*Oreochromis niloticus*	Actinopteri	Cichliformes	Cichlidae	*Bacillus subtilis* and *Bacillus licheniformis*	Probiotic	No	[75]
*Oreochromis niloticus*	Actinopteri	Cichliformes	Cichlidae	*Metschnikowia sp.* GXUS03	Probiotic	No	[76]
*Sparus aurata*	Actinopteri	Spariformes	Sparidae	Sodium butyrate	Butyrate	455 GS FLX Titanium, Amplicon: V1–V3	[77]
*Seriola lalandi*	Actinopteri	Carangiformes	Carangidae	Oxytetracycline, erythromycin and metronidazole	Antibiotic	Illumina MiSeq, Amplicon: V1–V2	[78]
*Piaractus mesopotamicus*	Actinopteri	Characiformes	Serrasalmidae	Florfenicol	Antibiotic	Illumina MiSeq, Shotgun metagenome	[79]
*Channa striata*	Actinopteri	Anabantiformes	Channidae	β-glucan, galactooligosaccharides, mannan-oligosaccharide	Prebiotic	T-RFLP fragment sequencing, Amplicon	[80]
*Channa striata*	Actinopteri	Anabantiformes	Channidae	*Saccharomyces cerevisiae* and *Lactobacillus acidophilus*	Probiotic	T-RFLP fragment sequencing, Amplicon	[80]
*Cyprinus carpio*	Actinopteri	Cypriniformes	Cyprinidae	Chinese yam peel	Plant	Illumina MiSeq, Amplicon: V3–V4	[81]
*Lates calcarifer*	Actinopteri	Perciformes	Centropomidae	Sodium diformate	Formate	No	[82]
*Oreochromis niloticus*	Actinopteri	Cichliformes	Cichlidae	*Bacillus subtilis* and *Lactobacillus plantarum*	Probiotic	ABI PRISM 377 sequencer (Perkin-Elmer), Amplicon: V6–V8	[83]
*Sparus aurata*	Actinopteri	Spariformes	Sparidae	Poultry by-product meal and Hydrolyzed feather meal	Protein	455 GS FLX Titanium, Amplicon: V3–V4	[84]
*Sparus aurata*	Actinopteri	Spariformes	Sparidae	Fish protein hydrolysate or Autolysed dried yeast	Protein	Illumina MiSeq, Amplicon: V3–V4	[85]
*Dicentrarchus labrax*	Actinopteri	Perciformes	Moronidae	Galactomannan oligosaccharides and A mixture of garlic and labiatae-plants oils	Prebiotic	Illumina MiSeq, Amplicon: V3–V4	[86]
*Salmo salar*	Actinopteri	Salmoniformes	Salmonidae	*Pediococcus acidilactici* MA18/5M and Short chain fructooligosaccharides	Synbiotic	Amplicon: V3	[87]
*Arapaima gigas*	Actinopteri	Osteoglossiformes	Osteoglossidae	*Lactococcus lactis* subsp. *lactis* and *Enterococcus faecium*	Probiotic	Amplicon: V1–V2	[88]
*Cyprinus carpio*	Actinopteri	Cypriniformes	Cyprinidae	Dietary plant proteins	Protein	Illumina HiSeq, Amplicon: V3–V4	[89]
*Carassius auratus*	Actinopteri	Cypriniformes	Cyprinidae	*Bacillus subtilis* and *Enterococcus faecium*	Probiotic	Amplicon: V3–V4	[90]
*Totoaba macdonaldi*	Actinopteri	Perciformes	Sciaenidae	Commercial dietary prebiotic and probiotic	Synbiotic	Illumina MiSeq, Amplicon: V3–V4	[91]
*Totoaba macdonaldi*	Actinopteri	Perciformes	Sciaenidae	Soy protein concentrate	Protein	Illumina MiSeq, Amplicon: V3–V4	[92]

**Table 2:** The studies on the gut microbiome of hybrid fish.

Host/Parents	Class	Order	Family	NGS Platform	Amplicon Sequencing	Reference
*Culter alburnus* ♀ × *Megalobrama amblycephala* ♂	Actinopteri	Cypriniformes	Xenocyprididae	Illumina MiSeq	Amplicon: V3–V4	[39]
*Parachondrostoma toxostoma/Chondrostoma nasus*	Actinopteri	Cypriniformes	Leuciscidae	Illumina MiSeq	Amplicon: V4	[48]
*Epinephelus fuscoguttatus* ♀ × *E. lanceolatus* ♂	Actinopteri	Perciformes	Serranidae	Illumina NovaSeq	Amplicon: V3–V4	[93]
*Epinephelus fuscoguttatus* ♀ × *E. lanceolatus* ♂	Actinopteri	Perciformes	Serranidae	Unknown	Unknown	[94]
*Oreochromis niloticus* ♀ × *O. aureus* ♂	Actinopteri	Cichliformes	Cichlidae	Unknown	Amplicon: V3	[95]
*Acipenser baerii* × *A. schrenckii*	Actinopteri	Acipenseriformes	Acipenseridae	Illumina HiSeq	Amplicon: V3–V4	[96]
*Pangasianodon gigas* × *Pangasianodon hypophthalmus*	Actinopteri	Siluriformes	Pangasiidae	Unknown	Unknown	[97]
*Epinephelus fuscoguttatus* ♀ × *E. lanceolatus* ♂	Actinopteri	Perciformes	Serranidae	Illumina HiSeq	Amplicon: V3–V4	[98]
*Acipenser baeri Brandt* ♀ × *A. schrenckii Brandt* ♂	Actinopteri	Acipenseriformes	Acipenseridae	Illumina MiSeq	Amplicon: V3–V4	[99]
*Epinephelus fuscoguttatus* ♀ × *E. lanceolatus* ♂	Actinopteri	Perciformes	Serranidae	Illumina HiSeq	Amplicon: V3–V4	[100]
*Epinephelus fuscoguttatus* ♀ × *E. lanceolatus* ♂	Actinopteri	Perciformes	Serranidae	Lon GeneStudio S5™	Amplicon: V4	[101]
*Epinephelus fuscoguttatus* ♀ × *E. lanceolatus* ♂	Actinopteri	Perciformes	Serranidae	Illumina	Amplicon: V3–V4	[102]
*Epinephelus fuscoguttatus* ♀ × *E. lanceolatus* ♂	Actinopteri	Perciformes	Serranidae	Illumina	Amplicon: V3–V4	[103]
*Epinephelus fuscoguttatus* ♀ × *E. lanceolatus* ♂	Actinopteri	Perciformes	Serranidae	Illumina MiSeq	Amplicon: V3–V4	[104]
*Morone chrysops* × *M. saxatilis*	Actinopteri	Perciformes	Moronidae	Illumina MiSeq	Amplicon: V1–V3	[105]
*Coregonus*	Actinopteri	Salmoniformes	Salmonidae	Illumina MiSeq	Amplicon: V3–V4	[106]
*Hypophthalmichthys nobilis* × *H. molitr*	Actinopteri	Cypriniformes	Xenocyprididae	Illumina MiSeq	Amplicon: V4	[107]
*Epinephelus fuscoguttatus* ♀ × *E. lanceolatus* ♂	Actinopteri	Perciformes	Serranidae	Illumina MiSeq	Amplicon: V3–V4	[108]
*Epinephelus fuscoguttatus* ♀ × *E. lanceolatus* ♂	Actinopteri	Perciformes	Serranidae	Unknown	Amplicon	[109]
*Epinephelus fuscoguttatus* ♀ × *E. lanceolatus* ♂	Actinopteri	Perciformes	Serranidae	Illumina MiSeq	Amplicon	[110]
*Morone Chrysops* × *M. Saxatilis*	Actinopteri	Perciformes	Moronidae	Illumina MiSeq	Amplicon: V1–V3	[111]
*Oreochromis niloticus* ♀ × *O. aureus* ♂	Actinopteri	Cichliformes	Cichlidae	454 Sequencer F.L.X.	Amplicon: V6–V8	[112]
*Oreochromis niloticus* ♀ × *O. aureus* ♂	Actinopteri	Cichliformes	Cichlidae	Unknown	Amplicon: V4	[113]
*Tachysurus fulvidraco* ♀ × *Pseudobagrus vachellii* ♂	Actinopteri	Siluriformes	Bagridae	Illumina MiSeq	Amplicon	[114]
*Epinephelus fuscoguttatus* ♀ × *E. lanceolatus* ♂	Actinopteri	Perciformes	Serranidae	Illumina	Amplicon: V3–V4	[115]
*Acipenser baerii* × *A. schrenckii*	Actinopteri	Acipenseriformes	Acipenseridae	Illumina HiSeq	Amplicon: V3–V4	[116]
*Epinephelus moara* ♀ × *E. lanceolatus* ♂	Actinopteri	Perciformes	Serranidae	Illumina HiSeq	Amplicon: V3–V4	[117]
*Epinephelus fuscoguttatus* ♀ × *E. lanceolatus* ♂	Actinopteri	Perciformes	Serranidae	Illumina MiSeq	Amplicon: V3–V4	[118]
*Acipenser baerii* × *A. schrenckii*	Actinopteri	Acipenseriformes	Acipenseridae	Illumina MiSeq	Amplicon: V3–V4	[119]
*Epinephelus fuscoguttatus* ♀ × *E. lanceolatus* ♂	Actinopteri	Perciformes	Serranidae	Illumina HiSeq	Amplicon: V3–V4	[120]
*Epinephelus fuscoguttatus* ♀ × *E. lanceolatus* ♂	Actinopteri	Perciformes	Serranidae	Illumina HiSeq	Amplicon	[121]
*Epinephelus fuscoguttatus* ♀ × *E. lanceolatus* ♂	Actinopteri	Perciformes	Serranidae	Unknown	Amplicon	[122]
*Epinephelus fuscoguttatus* ♀ × *E. lanceolatus* ♂	Actinopteri	Perciformes	Serranidae	Illumina HiSeq	Amplicon	[123]
*Acipenser baerii* × *A. schrenckii*	Actinopteri	Acipenseriformes	Acipenseridae	Illumina HiSeq	Amplicon: V3–V4	[124]

Unknown, the information is unclear in the reference.
